# *GNAi2/gip2*-Regulated Transcriptome and Its Therapeutic Significance in Ovarian Cancer

**DOI:** 10.3390/biom11081211

**Published:** 2021-08-14

**Authors:** Ji Hee Ha, Muralidharan Jayaraman, Mingda Yan, Padmaja Dhanasekaran, Ciro Isidoro, Yong Sang Song, Danny N. Dhanasekaran

**Affiliations:** 1 Stephenson Cancer Center, The University of Oklahoma Health Sciences Center, Oklahoma City, OK 73104, USA; Jihee-ha@ouhsc.edu (J.H.H.); Muralidharan-jayaraman@ouhsc.edu (M.J.); Mingdaok@gmail.com (M.Y.); Padmaja-dhanasekaran@ouhsc.edu (P.D.); 2 Department of Cell Biology, The University of Oklahoma Health Sciences Center, Oklahoma City, OK 73104, USA; 3 Laboratory of Molecular Pathology and NanoBioImaging, Department of Health Sciences, Università del Piemonte Orientale, 28100 Novara, Italy; ciro.isidoro@med.uniupo.it; 4 Department of Obstetrics and Gynecology, Cancer Research Institute, College of Medicine, Seoul National University, Seoul 151-921, Korea; yssong@snu.ac.kr

**Keywords:** ovarian cancer, *GNAi2*, *gip2*, transcriptome, gene expression, bio-informatics

## Abstract

Increased expression of *GNAi2*, which encodes the α-subunit of G-protein i2, has been correlated with the late-stage progression of ovarian cancer. *GNAi2*, also referred to as the proto-oncogene *gip2*, transduces signals from lysophosphatidic acid (LPA)-activated LPA-receptors to oncogenic cellular responses in ovarian cancer cells. To identify the oncogenic program activated by *gip2*, we carried out micro-array-based transcriptomic and bioinformatic analyses using the ovarian cancer cell-line SKOV3, in which the expression of *GNAi2*/*gip2* was silenced by specific shRNA. A cut-off value of 5-fold change in gene expression (*p* < 0.05) indicated that a total of 264 genes were dependent upon *gip2*-expression with 136 genes coding for functional proteins. Functional annotation of the transcriptome indicated the hitherto unknown role of *gip2* in stimulating the expression of oncogenic/growth-promoting genes such as KDR/VEGFR2, CCL20, and VIP. The array results were further validated in a panel of High-Grade Serous Ovarian Carcinoma (HGSOC) cell lines that included Kuramochi, OVCAR3, and OVCAR8 cells. Gene set enrichment analyses using DAVID, STRING, and Cytoscape applications indicated the potential role of the *gip2*-stimulated transcriptomic network involved in the upregulation of cell proliferation, adhesion, migration, cellular metabolism, and therapy resistance. The results unravel a multi-modular network in which the hub and bottleneck nodes are defined by ACKR3/CXCR7, IL6, VEGFA, CYCS, COX5B, UQCRC1, UQCRFS1, and FYN. The identification of these genes as the critical nodes in *GNAi2/gip2* orchestrated onco-transcriptome establishes their role in ovarian cancer pathophysiology. In addition, these results also point to these nodes as potential targets for novel therapeutic strategies.

## 1. Introduction

Despite major advances in anticancer drug development research and newer treatment modalities, ovarian cancer lags behind other cancers by failing to show a significantly improved survival rate over the years [1,2]. This is primarily due to the late diagnosis of the disease, which is further compounded by the therapy resistance of the recurrent disease [3,4]. While targeted therapy is emerging as an important deterrent to overcome drug resistance in many cancers including ovarian cancer, it requires a better understanding of the disease mechanism and causative factors involved in disease progression [4,5]. Plasticity of tumor cells, as well as pathway-bypass mechanisms involving the expression and/or activation of surrogate signaling molecules, were observed to blunt the effectiveness of an otherwise proven targeted therapy [6,7]. In this context, it is of interest to note that the α-subunit of G protein i2, which is encoded by the gene *GNAi2* and often referred to as *gip2* proto-oncogene, shows a biased increased expression in the late stages of ovarian cancer [8]. The role of *gip2* in ovarian cancer pathobiology becomes all the more important considering the fact that GNAi2 is activated by lysophosphatidic acid (LPA), an endogenous growth factor in ovarian cancer. Such a context-specific expression of *gip2* along with its potent role in activating diverse oncogenic signaling responses such as cell proliferation, EMT, stemness, and invasive metastatic migration necessitates a need to define the global transcriptomic changes associated with the expression of *gip2* so that potential therapeutic nodes can be identified. With this rationale, we sought to gain insight into the onco-transcriptome stimulated by *gip2* in ovarian cancer cells and its functional role in ovarian cancer progression and/or therapy resistance. We report the results from micro-array-based transcriptomic analysis and gene set enrichment analysis on defining *gip2*-dependent transcriptomic network along with the associated hub and bottleneck genes in ovarian cancer cells.

Transcriptome profiling of SKOV3 cells in which the expression of *gip2* was silenced indicated the *gip2*-dependent expression of 264 genes, of which 136 were found to be coding for functional proteins. Many of these genes, totaling 78, are known to be associated with the hallmarks of cancer. The array results were validated by monitoring the expression of *KDR/VEGFR2*, *CCL20*, and *VIP*, as a representative set of pro-tumorigenic genes in the high-grade serous ovarian carcinoma cell lines Kuramochi and OVCAR8. Gene Ontology (GO) enrichment and protein–protein interaction (PPI) network analyses of the dataset from the transcriptome were carried out using the web-based Database for Annotation, Visualization and Integrated Discovery (DAVID), Search Tool for Retrieval of Interacting Genes (STRING), and Cytoscape applications. Gene enrichment analysis indicated the oncogenic role of *gip2* in inducing the expression of genes involved in cell proliferation, adhesion, and migration. PPI network analysis identified the critical pathways regulated by the transcriptome, which include cellular energetics, oncogenic signaling, and therapy resistance. Further network analysis using Cytoscape application identified the potential pro-tumorigenic role of hub and bottleneck genes, namely *CYCS*, *VEGFA*, *IL6*, *UQCRFS1*, *UQCRC1*, *COX5B*, *ACKR3*/*CXCR7*, and *FYN*, in *gip2*-orchestrated onco-transcriptome. Functional annotation of the hub and bottleneck genes indicated a triplex signaling mode driving ovarian cancer progression. This involves the activation of a network cluster that plays a stimulatory role in cancer cell metabolism, cell proliferation, and invasive migration. In addition to providing new insights into the network organization of *gip2*-stimulated onco-transcriptome in ovarian cancer, the results provide a molecular basis for investigating the therapeutic potential of the hub and bottleneck nodes such as those of *UQCRFS1*, *ACKR3*/*CXCR7*, and *FYN* for the development of second-line targeted therapy in ovarian cancer.

## 2. Materials and Methods

### 2.1. Cell Lines and Culture

High-grade serous carcinoma cell lines OVCAR3 and non-serous ovarian carcinoma cell line SKOV3 were acquired from American Type Culture Collection (ATCC, Manassas, VA, USA), OVCAR8 and Kuramochi cells were procured from the National Cancer Institute (NCI, Bethesda, MD, USA) and Japanese Collection of Research Biosources Cell Bank (JCRB, Osaka, Japan) respectively. Routine authentication of the cell lines was carried out by short tandem repeat analysis as described [9]. Cell-culture conditions and the use of SKOV3 cell lines expressing shRNAs targeting *GNAi2/gip2* and non-targeting scrambled shRNA were previously described [10]. OVCAR3, OVCAR8, and Kuramochi cells were maintained in Roswell Park Memorial Institute (RPMI) 1640 medium (Cellgro, Manassas, VA, USA) whereas SKOV3 cells were cultured in Dulbecco’s modified Eagle’s (DMEM) Medium (Cellgro, Manassas, VA, USA) supplemented with 10% FBS (Gemini Bio-Products, West Sacramento, CA, USA), 50 U/mL penicillin, 50 μg/mL streptomycin (Cellgro, Manassas, VA, USA) at 37 °C and 5% CO_2_. siRNAs targeting *GNAi2/gip2* (siGENOME Human *GNAi2* siRNA SMARTpool; Cat # M-008435-00-0005) and non-targeting scrambled control siRNAs control (siGENOME Non-Targeting siRNA Pool; Cat # D-001206-13-05) were purchased from Dharmacon/Horizon Discovery, Lafayette, CO. *GNAi2/gip2*-specific siRNA and siCon were transfected into Kuramochi, OVCAR3 and OVCAR8 cells using Lipofectamine RNAiMAX (Invitrogen, Life Technologies, Carlsbad, CA, USA) as recommended by the manufacturer. The knockdown of *gip2* in the transfectants was confirmed using RT-PCR. LPA (1-oleoyl-2-hydroxy-sn-glycero-3-phosphate) used in the study was prepared as 10 mM stock solution in PBS containing 1% BSA and stored at −80 °C until use.

### 2.2. Transcriptomic Analysis

Serum-starved stable SKOV3-shScr (nonspecific scrambled shRNA control) and SKOV3-sh*gip2* cells were stimulated with LPA (10 μM) for 16 h. Qiagen RNeasy mini kit (Qiagen, Carlsbad, CA) was used to extract total RNA following the manufacturer’s protocol. Agilent SurePrint G3 Human Comparative Genomic Hybridization 8 × 60 microarray platform was employed to generate the transcriptomic profile of these stable cell lines. Complementary RNAs were labeled with Cy3-CTP using the Agilent Quick Amp labeling kit (Agilent, CA) and hybridized to the array slides following the manufacturer’s protocol. A total of six samples (3 control and 3 experimental) were used in the microarray platform. Agilent SureScan scanner was used to scan the array slides at 2 microns resolution and the spot intensity extracted using Agilent Feature Extraction version 11.0 software. Delineation of gene expression between the cell lines was established using Agilent GeneSpring GX version 13.0. *gip2*-dependent genes, with ≥ a 5-fold decrease over the control cells, were used for further bioinformatic analyses.

### 2.3. Bioinformatic Analysis

Multiple web-based enrichment and network analysis were employed to define the pathways and network their interactions. Database for Annotation, Visualization and Integrated Discovery (DAVID) annotation tool (https://david.ncifcrf.gov/home.jsp) (accessed on 21 July 2021) was used for the Gene Ontology Enrichment analysis [11]. Protein–protein interaction (PPI) network analysis was carried out employing the web-based Search Tool for the Retrieval of Interacting Genes/Proteins (STRING) database (https://string-db.org/) (accessed on 21 July 2021) with the high confidence interaction score (0.7) and <10 degrees of interaction limit [12]. Cytoscape software application was used to identify the significant modules in the PPI network [13]. The hub and bottleneck nodes of the PPI network were identified using the cytoHubba plugin in Cytoscape [14]. While the hub nodes of the PPI network were identified using Degree, MCC, MNC, EPC, EcCentricity, Closeness, Betweenness, and Clustering Coefficient algorithms, the bottleneck nodes were identified using the BottleNeck algorithm of the cytoHubba plugin [15].

### 2.4. RT-qPCR Analysis

Total RNA was extracted using Qiagen RNeasy kit (Qiagen, Valencia, CA, USA) following the manufacturer’s instructions. cDNA synthesis was carried out using iScript™ cDNA Synthesis Kit (BioRad, Hercules, CA, UAS). Real-time quantitative PCR (RT-qPCR) was carried out using the cDNA from the above step using the SsoAdvanced Universal SYBR Green Supermix (BioRad, Hercules, CA, USA) in a BioRad CFX96 Real-time PCR detection system. The raw Cq values were normalized against GAPDH, a housekeeping gene. The primers used in this study are shown in Table S1.

### 2.5. Immunoblot Analysis

Immunoblot Analysis. Antibodies to GNAi2 (sc-409), GAPDH (CB1001), peroxidase-conjugated anti-rabbit IgG (W401B) were from Santa Cruz Biotechnology Inc (Dallas, TX, USA), Abcam (Cambridge, MA, USA) and Promega Corporation (Madison, WI, USA) respectively. Immunoblot analysis was carried out using our previously published methods [10].

### 2.6. Statistics

All gene expression studies were tested by a two-tailed Student’s t-test with Welch’s correction using GraphPad Prism software (La Jolla, CA, USA). *p* values and False Discovery Rates in the bioinformatic analysis were from built-in statistical analytical tools within respective programs.

## 3. Results

### 3.1. Characterization of GNAi2/gip2-Dependent Transcriptome

**Figure 1:**
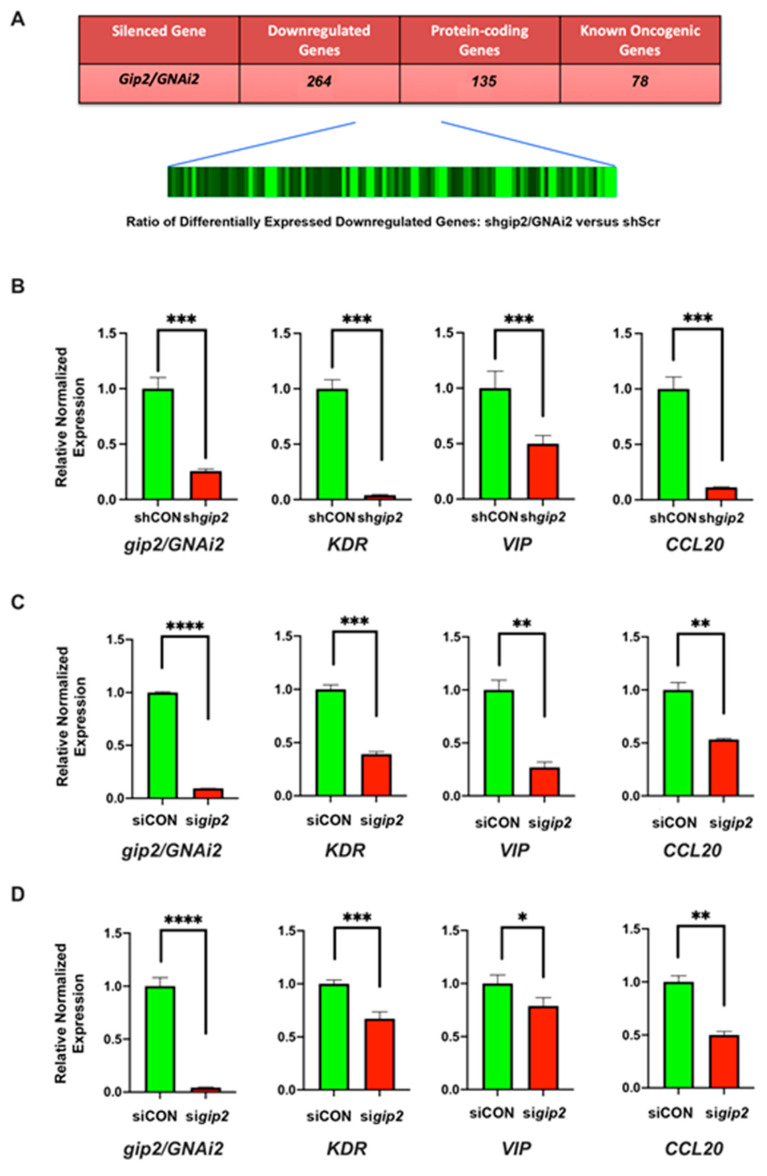
Heatmap of genes downregulated upon silencing of GNAi2/gip2 and validation. (**A**) Heatmap and the downregulated genes. The ratio comparing control cells (scrambled shRNA) and gip2-silenced cells is presented as a heat map. Black and green bands represent unchanged and downregulated expression, respectively. Total number of genes that show > 5-fold reduction in the expression compared to control values is presented as a table insert. *Array results were* validated by RT-PCR methods using SKOV3 (**B**) Kuramochi (**C**) and OVCAR8 (**D**) cells in which the expression of gip2 was silenced (sigip2) using specific shRNA or siRNA. In SKOV3 cells, cells stably expressing scrambled shRNA (shCON) were compared with cells in which gip2/GNAi2 was silenced with the stable expression of specific shRNA targeting gip2/GNAi2 (shgip2). In Kuramochi and OVCAR8 cells, cells transfected with non-targeting scrambled siRNA pool were used as the control group (siCON). Downregulated genes were validated by monitoring the expression of the representative genes KDR, VIP, and CCL20 by RT-PCR. Statistical significance between gip2-knockdown and scrambled siRNA cells was determined by Student’s *t*-test (* *p* < 0.05, ** *p* < 0.005, *** *p* < 0.0005, **** *p* < 0.0001ACKR3).

Our previous studies have shown that LPA-LPAR activation or mutational activation of GNAi2/gip2 stimulates proliferation, EMT, invasive migration, and metabolic reprogramming of ovarian cancer cells [9,16,17,18,19]. To gain better insight into the global oncogenic network regulated by gip2, we carried out transcriptome profiling of SKOV3 ovarian cancer cells in which the expression of gip2 was silenced using shRNAs [10]. The cells were stimulated with LPA and the genes that showed a decrease in the gip2-silenced cells compared to the scrambled shRNA control group were identified using Agilent array. With the fold change cut-off value of ≥5 compared to control cells, gip2-silenced cells showed a downregulation of 264 genes (Figure 1A; GEO Accession No: GSE173214). Of the downregulated genes, only 135 genes were found to be protein-encoding genes (Table S2). Others were represented by genes encoding uncharacterized transcripts or the ones encoding pseudogenes, anti-sense RNAs, or non-coding RNAs (GEO Accession No: GSE173214). Analysis of the genes through data mining from published literature indicated that 78 of these genes are known to play an oncogenic role in different cancers (Table S3). More interestingly, querying these genes in TCGA ovarian cancer dataset (TCGA, Firehose Legacy) via CBioPortal, indicated that 61 of these genes showed increased expression in ovarian cancer (Table S4) and 40 of these genes showed co-occurrence in their expression profiles (Table S5), thus further validating our results in ovarian cancer patient subgroup. These array results were experimentally validated by monitoring the expression of a representative set of pro-tumorigenic genes in the SKOV3 cell line and high grade serous ovarian carcinoma (HGSOC) cell lines Kuramochi and OVCAR8. Expression of gip2 was silenced using shRNA (SKOV3 cells) or siRNAs specifically targeting gip2 (Kuramochi and OVCAR8 cells). Expression of KDR/VEGFR2, VIP, and CCL20-a representative set of genes from the array results-were monitored by RT-PCR. As shown in Figure 1B–D, silencing of gip2 decreased the expression of all these genes in SKOV3 as well as Kuramochi and OVCAR8 cells, thus validating the array results.

### 3.2. Gene Ontology Enrichment Analysis of gip2-Dependent Genes

In the gip2-silenced cellular model system used here, the genes that show decreased expression upon the silencing of gip2-the gip2-dependent genes-are in fact defined as the genes that would be stimulated by gip2 in situ in ovarian cancer cells. Consistent with this premise, functional annotation of these genes indicated that at least 50% of the genes (a total of 78 genes out of 136 protein-coding genes) were found to play an oncogenic role in different cancers (Table S2). Reasoning that the functional networking of these genes could provide insight into the mechanism by which gip2 promotes neoplastic growth of ovarian cancer cells, we carried out pathway and network analyses. Since Gene Ontology (GO) enrichment analysis could provide information on the functional relationship among a large set of genes, GO analysis of biological processes (GO:BP), molecular functions (GO:MF) and cellular components (GO:CC) were carried out using DAVID database [11]. In GO:BP ontology, the gip2-dependent genes were significantly enriched in biological processes involving cell adhesion, proliferation, and cell motility (Table 1). In GO:CC ontology, cellular periphery including plasma membrane and membrane region formed the major categories. In GO:MF ontology, the topmost enriched categories were molecular transducer activity, receptor signaling activity, and lipid and tyrosine kinase binding (Table 1).

### 3.3. Analysis of PPI Networks and Pathways

**Figure 2:**
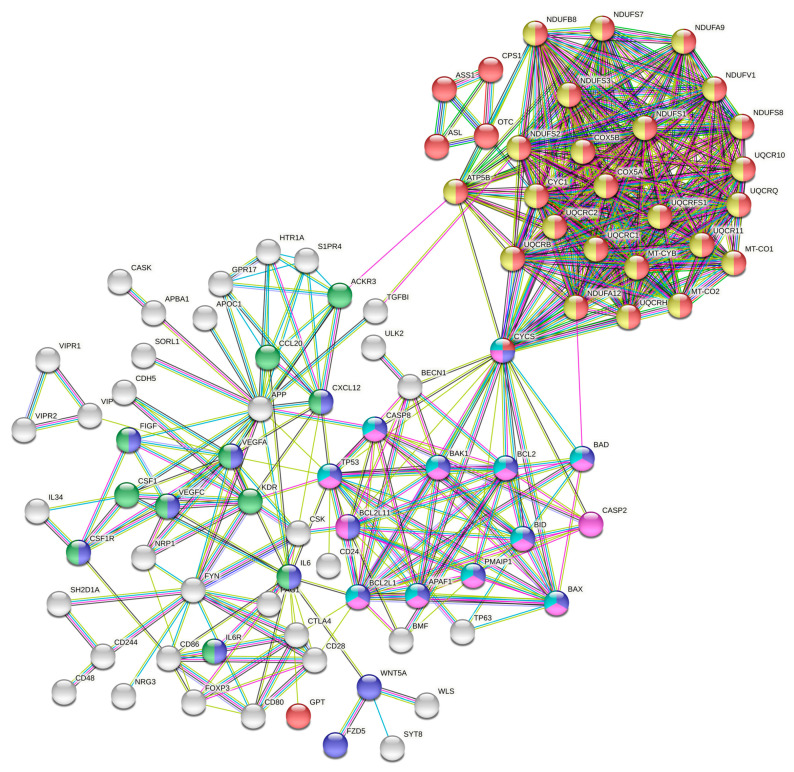
Protein–Protein Interaction Network of gip2-dependent genes.

To further investigate the functional interactions among the proteins encoded by the gip2-dependent genes, downregulated in gip2-silenced cells, we carried out Protein–Protein Interaction Networks Functional Enrichment Analysis using STRING database [12]. Gip2-dependent genes were analyzed with a high confidence interaction score (0.7) and < 10 degrees of interaction. The PPI network was constructed by screening 185 nodes and 473 edges (Figure 2). The most significant modules in the PPI network were determined using the Cytoscape software application [13].

Using the web-based STRING tool, the PPI network of the genes downregulated in gip2-silenced cells was constructed. Query proteins and their first shell interactions are denoted by colored nodes. Second shell interactions are in while. Key pathway clusters defined by specific nodes are denoted by different colors as follows: Red, metabolic pathway; Blue, Pathways in Cancer; Green, Cytokine–Cytokine Receptor Interactions; Yellow, Oxidative Phosphorylation; Violet, apoptosis; and Aqua Marine, Platinum Resistance. Predicted functional interactions are indicated by the connecting lines. The colors of the lines represent the types of evidence that were used to predict the PPI associations. They are as follows: Red-known gene fusions; Green-gene neighborhood; Blue-gene co-occurrence; Purple-experimental data; Yellow-text-mining; Light Blue-protein homology; Aqua Marine-curated database; and Black-co-expression.

KEGG analyses indicated that the top pathways defined by the gip2-dependent genes were: 1. Metabolic pathways; 2. Oxidative phosphorylation; 3. Pathways in cancer; 4. Platinum resistance; and 5. EGFR-inhibitor resistance (Table 2). Reactome analysis expanded this further into pathways associated with 1. Respiratory electron transport; 2. Apoptosis; 3. Hemostasis; 4. Mitochondrial protein support; and 5. Interleukin-6 signaling (Table 2).

### 3.4. Identification of the Hub and Bottleneck Nodes

We probed the network further to identify the critical genes that define the hub nodes of the network using the cytoHubba plugin in Cytoscape [13]. We applied the multiple algorithms of the cytoHubba including Degree, MCC, MNC, EPC, EcCentricity, Closeness, Betweenness, and Clustering Coefficient to identify the hub nodes of the PPI networks [14]. The intersecting genes identified by the different algorithms were tabulated. Results from this analysis identified CYC5, VEGFA, COX5B, UQCRFS1, and IL6 as the top five hub nodes of the network (Table 3). In addition, we identified the bottleneck nodes of the network since they are considered as the “connector” or “choke points” in PPI networks [19,20] using the BottleNeck algorithm in cytoHubba application of Cytoscape. The results identified CYCS, VEGFA, and IL6 along with ACKR3/CXCR7 and FYN as the top five bottleneck node genes of the network (Table 3). Functional annotation of these genes points to the diverse oncogenic roles of these genes in many different cancers including ovarian cancer (Table 3).

**Figure 3:**
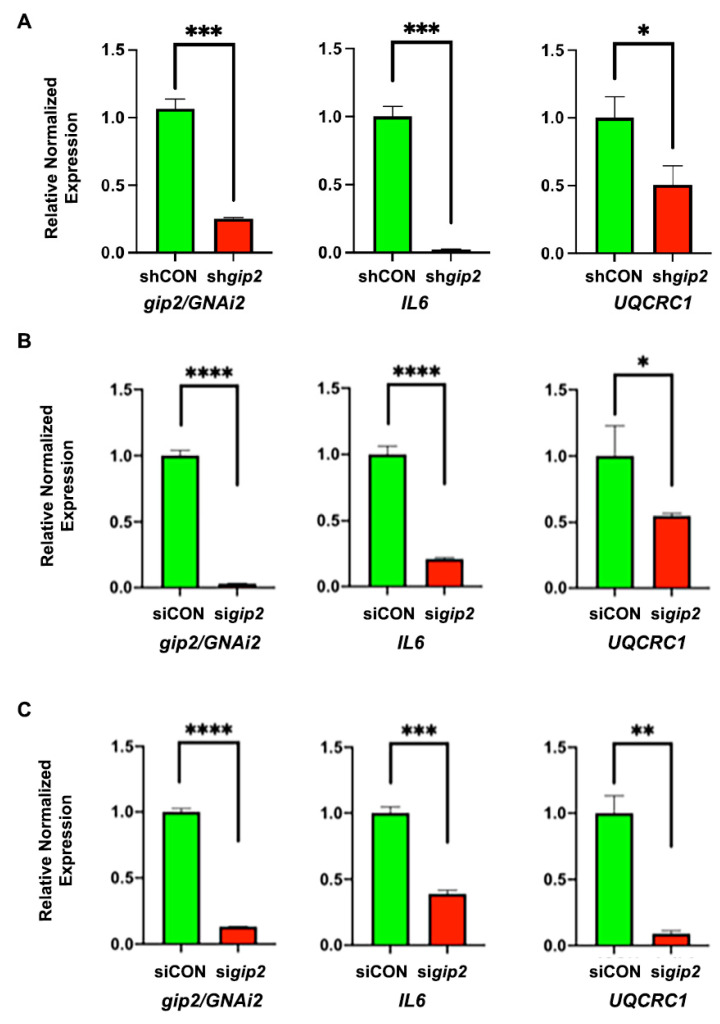
Validation of gip2-dependent hub and bottleneck genes. Downregulation of Hub and Bottleneck node genes upon silencing of gip2 was validated using SKOV3 (**A**), OVCAR8 (**B**) and OVCAR3 (**C**) cells in which the expression of gip2 was silenced using specific shRNA (SKOV3) or siRNAs (OVCAR8 and OVCAR3). In the case of SKOV3 cells, cells expressing non-targeting scrambled RNA were used as control group (shCON). In OVACR8 and OVCAR3 cell, cells transfected with non-targeting scrambled siRNA pool were used as the control group (SiCON). Downregulated genes were validated by monitoring the expression of the representative bottleneck gene IL6 and hub gene UQCRC1. Statistical significance was determined by Student’s *t*-test (* *p* < 0.05, ** *p* < 0.005, *** *p* < 0.0005, **** *p* < 0.0001).

Gip2-dependent expression of the hub and bottleneck genes were validated by monitoring the expression of IL6 and UQCRC1 in gip2-silenced SKOV3, OVCAR8 and OVCAR3 cells by RT-PCR analysis. As shown in Figure 3, gip2-silencing led to the decreased expressions of both IL6 and UQCRC1 in all the tested cell lines (Figure 3).

### 3.5. Significance of the Hub and Bottleneck Nodes

**Figure 4:**
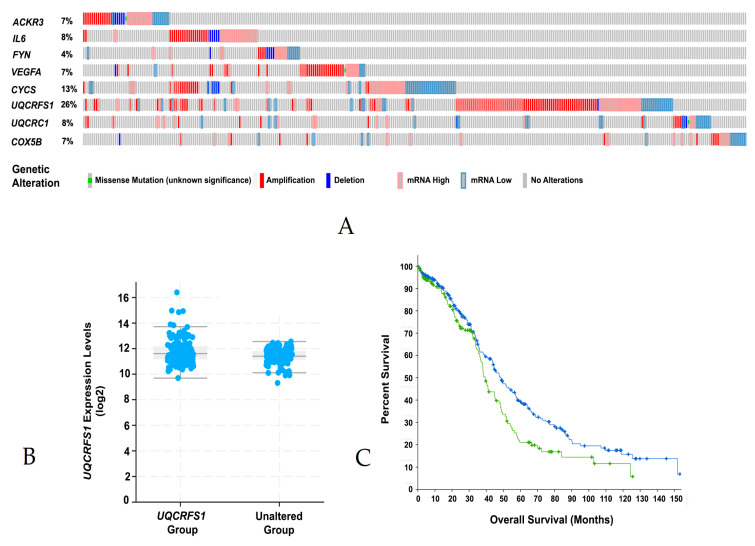
Genomic and expression profile of hub and bottleneck genes in Ovarian cancer patients. Genomic and expression profiles of the hub and bottleneck genes in ovarian cancer patients were visualized in OncoPrint at cBioPortal web-portal (**A**). Plots depicting the expression of the hub node gene UQCRFS1 with the *p*-value of 2.54e−8 were obtained from the CBioPortal (**B**). Overall survival of patients with UQCRFS1 amplification or overexpression (Log Rank Test *p*-value of 3.9e−2) was extracted from the CBioPortal (**C**).

Next, we investigated the biological significance of the hub and bottleneck node genes in relation to ovarian cancer through cBioPortal analysis [20,21]. Oncoprint profile of CYCS, VEGFA, IL6, UQCRC1, UQCRFS1, COX5B, ACKR3/CXCR7, and FYN indicated that these genes were either amplified or overexpressed in 4–25% of the ovarian cancer patients (Figure 4A). Increased amplification or expression seen with UQCRFS1 in ovarian cancer patients, prompted us to carry out in silico analysis of its expression profile and overall survival rate of the ovarian cancer patients who show the altered expression of UQCRFS1. RNASeq data and overall survival plot were obtained through CBioPortal analyses. As shown in Figure 4B, increased expression of UQCRFS1 (Figure 4B) correlated with the reduced overall survival of ovarian cancer patients (Figure 4C). More intriguingly, increased expression of gip2 was not observed in UQCRFS1-patients. It is possible that the initiating event involved in the expression of UQCRFS1 is the activation of gip2 rather than an increased expression of gip2 as in the case of other oncogenes [22]. Together with the functional annotation of the other hub and bottleneck genes, these results substantiate the potential role of the gip2-dependent hub/bottleneck nodal network in ovarian cancer pathophysiology.

## 4. Discussion

Effective targeted therapy in ovarian cancer has remained elusive primarily due to the heterogenous subtypes and aberrant signaling pathways associated with the disease [23,24]. While PARP inhibitors and anti-angiogenic agents have provided relief to many patients, the emergence of therapy resistance, especially in late-stage disease, has remained an unsurmountable clinical problem [25,26,27,28]. This has necessitated the critical need to have a clear understanding of the molecular events associated with different subtypes and stages of the disease so that context-specific therapeutic strategies can be developed. Although recent studies have identified major pathways and genetic risk factors associated with ovarian cancer [29], causative factors involved in sustaining tumor growth in the advanced stages of ovarian cancer are largely unknown. In this regard, the observation that *GNAi2/gip2* shows increased expression in advanced ovarian cancers is quite significant as it suggests the possibility that *gip2* could play a critical, if not unique, role in advanced ovarian cancers. Therefore, we investigated whether *gip2*-dependent transcriptome in ovarian cancer can be probed to identify any novel targets for ovarian cancer therapy. Results from such analysis, presented here, establish the critical role of the *gip2*-dependent transcriptomic network in pathways related to cell survival, proliferation, metastasis, adhesion, and cancer cell metabolism (Tables S2 and S3).

While the individual pathways activated by *gip2* have been cataloged in the past, the extent to which *gip2* is involved in regulating a comprehensive transcriptomic network to facilitate ovarian cancer progression has not been fully understood until now. Our results presented here provide the first evidence to show the synergistic signaling nodes regulated by *gip2* in promoting ovarian cancer growth (Table S2). Functionally, the transcriptomic nodes regulated by *gip2* range from pathways involved in cellular energetics to evading cell death. The genes validated in the HGSOC cells lines OVCAR8 and Kuramochi substantiate this point (Figure 1). *KDR* gene, which encodes vascular endothelial growth factor receptor 2, was shown to promote oncogenic signaling pathways in many different cancers including ovarian cancer [30,31]. Vasoactive Intestinal Peptide, encoded by *VIP*, is known to be involved in autocrine as well as paracrine signaling loop that promotes cancer growth in multiple cancer types [32]. *CCL20* gene encodes the chemokine C-C Motif Chemokine Ligand 20 and it was identified to play a role in metastasis and therapy resistance of ovarian cancer cells [33,34].

In addition to these genes, GO enrichment analysis of the data has identified several novel correlates associated with ovarian cancer growth and progression. Results from GO:CC enrichment analysis are in conformity with the known cellular and signaling locale of GNAi2 in transducing the signals from the membrane-bound LPAR when activated by LPA (Table 1). Similarly, the predicted molecular functions such as molecular transducer activity, signaling receptor activity, transmembrane signaling receptor activity, lipid binding, and protein tyrosine kinase binding, can all be related to the signal-transducing activity of *gip2* (Table 1). More interestingly, GO:BP analysis unravels certain novel aspects of *gip2*-regulated biological processes in ovarian cancer transcriptome. While the role of *gip2* in biological processes such as response to stimulus and intracellular signal transduction has been well characterized, gene enrichment in GO:BPs such as cell proliferation, adhesion, and migration provide a wider glimpse into the transcriptomic landscape activated by *gip2* in ovarian cancer cells. One of the characteristic features of late-stage ovarian cancer is the peritoneal dissemination of ovarian cancer cells, which precedes peritoneal and hematogenous metastasis of ovarian cancer [35]. Biological processes underlying peritoneal dissemination and subsequent metastasis involve cell migration, adhesion, and subsequent tumor angiogenesis [36]. While the molecular components of these pathways have been characterized to a certain extent, the integration of these pathways into a transcriptomic network and the master regulator that modulates the expression of the downstream signaling nodes have remained unknown. Results from the gene enrichment analyses point to such a regulatory role for *gip2* in the transcriptomic reprogramming in ovarian cancer cells.

Organizational features of the *gip2*-orchestrated transcriptomic network are more clearly discernible with the results from the PPI network analysis. Two major clusters can be identified in the PPI network: one that promotes cancer growth through multiple cancer-specific signaling pathways and the other that promotes cancer cell metabolism (Figure 2). The tumorigenic pathways cluster encompasses genes involved in cell proliferation, suppression of apoptosis, and platinum resistance. This cluster is further augmented by signaling circuits involving cytokines, chemokines, and their cognate receptors that are known to play a critical role in cancer cell proliferation, adhesion, invasive migration, and metastasis. The metabolism cluster, on the other hand, primarily includes the genes that encode proteins/enzymes involved in oxidative phosphorylation (Figure 2). Analyses of the hub and bottleneck signaling nodes of the PPI network provide further insight into the *gip2*-regulated transcriptome. The finding that the silencing of *gip2* leads to the reduced expressions of all of these hub and bottleneck genes underscores the critical roles of these nodes in the onco-transcriptome (Figure 3). Functional annotation of these hub and bottleneck genes as well as the Oncoprint analyses of these genes add further support to their tumorigenic roles in ovarian cancer (Table 3; Figure 4A).

**Figure 5:**
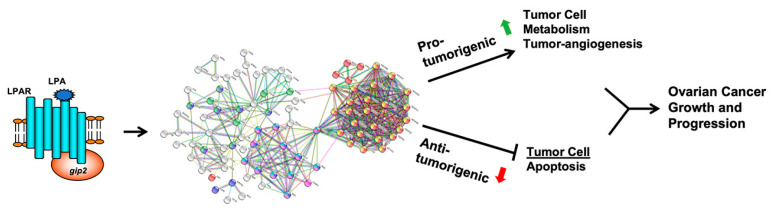
Gip2-regulated onco-transcriptome in ovarian cancer. LPA-LPAR-stimulation or mutational activation of *gip2* drove the coordinated regulation of two major network clusters. Gip2-stimulated pro-tumorigenic network comprised of nodes involved in tumor cell metabolism and angiogenesis synergizes with the suppression of anti-tumorigenic network consisting of pro-apoptotic and growth-suppressive nodes. LPA; lysophosphatidic acid; LPAR, lysophosphatidic acid receptor; *gip2*, G protein subunit α i2.

The known roles of *VEGFA*, *ACKR3*, *IL6* and *FYN* establish them as the major regulators of cancer pathways cluster in the network. Vascular Endothelial Growth Factor A, encoded by *VEGFA*, was found to be overexpressed in many cancers including ovarian cancer. *VEGFA* expression and its activation of VEGFRs were correlated to metastasis, tumor angiogenesis, and chemotherapy resistance in ovarian cancer [37,38,39]. Similarly, autocrine and paracrine signaling pathways stimulated by Interleukin-6, encoded by *IL6*, were shown to be associated with cancer cell proliferation, migration, stemness, and therapy resistance in ovarian cancer [40,41]. Likewise, the signaling pathways activated by CXCR7, a chemokine receptor encoded by *ACKR3*, were shown to be critically involved in the migration and invasive metastasis of multiple cancers [42,43]. Fyn kinase, encoded by *FYN*, stimulates a wide array of oncogenic pathways including cell proliferation, migration, EMT, and therapy resistance in many cancers [44,45,46]. Similarly, *CYCS*, *UQCRC1*, *UQCRFS1*, and *COX5B* genes present themselves as the regulatory nodes in the metabolism cluster of the network. Cytochrome C, encoded by *CYCS*, is a major component of the electron transport chain of mitochondria and it is tightly associated with the pro-survival pathways in normal as well as cancer cells [47]. *UQCRC1* encodes the mitochondrial ubiquinol-cytochrome c reductase core protein I, which is part of Complex I of the mitochondrial respiratory chain and was shown to be critically involved in the oncogenic reprogramming of metabolic pathways in pancreatic cancer [48]. *UQCRFS1*, another hub gene identified here, encodes the mitochondrial Ubiquinol-Cytochrome C Reductase/Rieske Iron-Sulfur polypeptide 1 and it is a key subunit of Complex III of the mitochondrial respiratory chain. Its overexpression and amplification were observed in different cancers including ovarian cancer [49,50,51,52]. Its overexpression was implicated in the aggressive phenotype of breast cancer [50,51]. *COX5B* gene encodes cytochrome c oxidase, and it is another critical component of the mitochondrial respiratory chain. Overexpression of *COX5B* is associated with a poor prognosis in many cancers [53,54]. The observation that these critical nodes are part of the gene cluster involved in mitochondrial oxidative phosphorylation gains further significance in light of the recent findings that mitochondrial oxidative phosphorylation is upregulated in many cancers including ovarian cancer [55,56,57]. Thus, the comprehensive analysis of the oncogenic network regulated by *gip2*indicates the activation of a transcriptomic network that involves cell metabolism, suppression of cell death, invasive metastasis, and tumor angiogenesis that cumulatively leads to aggressive ovarian cancer growth and progression (Figure 5).

It should be noted that the limitation of the results is that the transcriptome network depicted here is derived from the data using a single ovarian cancer cell line. Nevertheless, the findings that (1) Representative genes are validated experimentally in HGSOC cell lines (Figure 1 and Figure 3); (2) More than 50% of the genes in the network (78 genes) were shown to be dysregulated in many cancers including ovarian cancer (Table S3); (3) Analysis of these genes in TCGA ovarian cancer dataset indicated that 61 of these genes showed increased expression in ovarian cancer (Table S4); and (4). Forty of these genes showed co-occurrence in their expression profiles (Table S5) provide external validation of the potential role of these network genes in HGSOC cells and the ovarian cancer patient subgroup. The identification of CYCS, VEGFA, IL6, UQCRC1, UQCRFS1, COX5B, ACKR3/CXCR7, and FYN as pro-tumorigenic nodes designates them as the novel and potentially druggable targets for effective targeted adjuvant therapy for ovarian cancer. In this context, the role of *UQCRFS1* as a hub node warrants special mention. The query of this gene in cBioPortal indicated that *UQCRFS1* is either amplified or overexpressed in 26% of ovarian cancer patients (Figure 4A). More strikingly, the cBioPortal analysis indicates further that the aberrant expression of *UQCRFS1* could be correlated with the reduced overall survival of ovarian cancer patients (Figure 4B). While the therapeutic potentials of *VEGFA*, *ACKR3*, and *IL6* have been already investigated or exploited [43,58,59], our analysis presented here points to *UQCRSF1* as a, thus far unidentified, potential node for the development of novel therapeutics.

## 5. Conclusions

In summary, the results presented here provide a paradigm in which *GNAi2/gip2*-dependent transcriptome promotes aggressive cancer growth during advanced stages of ovarian cancer through the gene network that stimulates cell metabolism, invasive metastasis, tumor angiogenesis along with the suppression of cell death. Considering the late-stage expression profile of *GNAi2/gip2* in ovarian cancer, the hub and bottleneck nodes identified here, especially the metabolic signaling nodes such as *UQCRFS1*, should provide newer targets for the development of second-line targeted therapy for advanced ovarian cancers.

## Figures and Tables

**Table 1:** Gene ontogeny enrichment analysis of differentially expressed genes.

Category	Term	Description	Gene Count	*p*-Value
**GO: BP**	GO:0050896	Response to stimulus	67	2.9E−2
	GO:0009605	Intracellular signal transduction	25	7.1E−2
	GO:0042127	Regulation of cell proliferation	19	1.3E−1
	GO:0007155	Cell adhesion	17	9.5E−2
	GO:0016477	Cell migration	14	5.2E−2
**GO:CC**	GO:0005886	Plasma membrane	46	1.7E−2
	GO:0071944	Cell periphery	46	2.5E−2
	GO:0005576	Extracellular region	41	3.1E−2
	GO:0042995	Extracellular space	20	2.8E−3
	GO:0098862	Membrane region	6	8.5E−2
**GO:MF**	GO:0060089	Molecular transducer activity	18	3.8E−2
	GO:0038023	Signaling receptor activity	18	3.8E−2
	GO:0004888	Transmembrane signaling receptor activity	15	5.2E−2
	GO:0003982	Lipid binding	10	2.5E−2
	GO:0001228	Protein tyrosine kinase binding	3	5.5E−2

Gene ontology (GO) enrichment analysis was carried out with the genes downregulated in SKOV3 cells upon silencing of gip2 using the DAVID gene annotation tool. GO term enrichment analyses in terms of the three sub-ontologies: Biological Process (BP), Cellular Component (CC), and Molecular Function (MF) are presented.

**Table 2:** KEGG and Reactome pathway analysis of differentially expressed genes. KEGG and Reactome pathway enrichment analyses were carried out with the genes downregulated in gip2-silenced SKOV3 cells using STRING and Cytoscape applications. Major pathways of the PPI identified in downregulated and upregulated genes along with the gene count and false discovery rate are presented.

Pathway	Term	Description	Gene Count	False Discovery Rate
KEGG Pathway	hsa01100	Metabolic pathways	36	5.40e−08
	hsa00190	Oxidative phosphorylation	24	6.13e−21
	hsa05200	Pathways in cancer	21	5.83e−07
	hsa01524	Platinum drug resistance	11	5.45e−09
	hsa01521	EGFR tyrosine kinase inhibitor resistance	9	1.75e−06
Reactome Pathway	HSA-1428517	TCA Cycle and Respiratory electron transport	21	7.61e−16
	HSA-109581	Apoptosis	14	1.62e−08
	HSA-109582	Hemostasis	14	1.67e−02
	HSA-1268020	Mitochondrial protein import	4	2.76e−02
	HSA-1059683	Interleukin-6 signaling	2	3.69e−02

**Table 3:** Hub and bottleneck genes of the PPI network. The top five genes derived from the PPI network using MCC, MNC, Degree, EPC, and EcCentricity algorithms of the CytoHubba plugins in Cytoscape and top five bottleneck genes determined from the BottleNeck algorithm are presented.

Genes.	Nodes	Function	References
CYC5	Hub and Bottleneck	Increased oxidative phosphorylation and pro-survival cellular events	Huttemann et al., 2011
VEGFA	Hub and Bottleneck	Overexpression in ovarian cancer patients; tumor angiogenesis, associated with distant metastasis and resistance to chemotherapy	Guan et al., 2019; Sopo et al., 2019; Li et al., 2020
IL6	Hub and Bottleneck	Ovarian Cancer Growth, stemness, and therapy resistance	Wang et al., 2018; Azar et al., 2020
UQCRFS1	Hub	Oncogenic reprogramming of metabolism role in Pancreatic cancer	Kaneko et al., 2003; Ohashi et al., 2004; Owens et al., 2011; Jun et al., 2012
UQCRC1	Hub	Oncogenic reprogramming of metabolism role in Pancreatic cancer	Wang et al., 2020
COX5B	Hub	COX5B-mediated metabolic reprogramming is Associated with poor prognosis in many cancers	Gao et al., 2017; Chu et al., 2020
ACKR3/CXCR7	Bottleneck	Chemokine receptor activated in many cancers to promote invasive metastasis.	Neves et al., 2019; Smit et al., 2020
FYN	Bottleneck	Mediates oncogenic cell proliferation, migration, EMT, and therapy resistance in many cancers.	Saito et al., 2010; Lee et al., 2018; Yu et al., 2020

## Data Availability

The microarray data presented in the present study are deposited at the Gene Expression Omnibus (https://www.ncbi.nlm.nih.gov/geo/ accessed on 21 July 2021); accession no. GSE173214. Oncoprint data supporting the reported results used the Ovarian Serous Cystadenocarcinoma (TCGA, Firehose Legacy) dataset available at https://www.cbioportal.org (accessed on 21 July 2021).

## Supplementary Materials

The following are available online at https://www.mdpi.com/article/10.3390/biom11081211/s1, Table S1: RT-qPCR Primers; Table S2: *Gip2*-dependent Genes Downregulated upon Silencing of *gip2*; Table S3: Oncogenic Profile of gip2-dependent Genes; Table S4: Network Gene Alteration Frequency in Ovarian Cancer Patients; Table S5: Co-occurrence of Network Genes in Ovarian Cancer Patients.
