# Supplementary material for: GNAi2/gip2-Regulated Transcriptome and Its Therapeutic Significance in Ovarian Cancer
Source: Biomolecules. 2021 Aug 14;11(8):1211. doi: 10.3390/biom11081211 (PMC8393559; doi:10.3390/biom11081211)
Supplement: Supplementary file 1 [file biomolecules-11-01211-s001.zip › biomolecules-1329544-supplementary.pdf]

Table S1. *Reverse transcription-quantitative PCR primer sequences.*

Gene	Primers
CAGE1	FP: AAGCCCAGAGAAAAAGCCAGA<br>RP: ATGTACAAGTTATATGCCAACATGG
CCL20	FP: CCAAGAGTTTGCTCCTGGCT<br>RP: TGCTTGCTGCTTCTGATTCG
FYN	FP: TGACCTCCATCCCCAACTA<br>RP: TTCCCACCAATCTCCTTCC
Gip2	FP: AGCTGAGGACGAGGAGATGA<br>RP: GGATGATGGACGTGTCTGTG
IL6	FP: ACTCACCTCTTCAGAACGAATTG<br>RP: CCATCTTTGGAAGGTTCAGGTTG
KDR	FP: CTCTTGATCTGCCCAGGCTC<br>RP: GGCTCCAGTGTCATTTCCGA
UQCRC1	FP: GCCGGGGCACAAGTGCTAT<br>RP: CTTGGACAGCGCCTTGATGT
VIP	FP: CCAGGCATGCTGATGGAGTT<br>RP: CCCTCACTGCTCCTCTTTCC

Table S2. ***Gip2*-dependent Genes Downregulated upon Silencing of *gip2***

	GENE	Name	GenBank Accession
1.	*ABLIM1*	Actin binding LIM protein 1	NM_001003408
2.	*ACKR3*	Atypical chemokine receptor 3	NM_020311
3.	*AHNAK2*	AHNAK nucleoprotein 2	NM_138420
4.	*ANKDD1A*	Ankyrin repeat and death domain containing 1A	NM_182703
5.	*AOAH*	Acyloxyacyl hydrolase (neutrophil)	NM_001637
6.	*APBA1*	Amyloid beta (A4) precursor protein-binding, family A, member 1	NM_001163
7.	*APOC1*	Apolipoprotein C-I	NM_001645
8.	*ARHGEF28*	Rho guanine nucleotide exchange factor (GEF) 28	NM_001177693
9.	*ASS1*	Argininosuccinate synthase 1	NM_000050
10.	*ATAT1*	Alpha tubulin acetyltransferase 1	NM_024909
11.	*BAD*	BCL2-associated agonist of cell death	AK309150
12.	*BARX2*	BARX homeobox 2	NM_003658
13.	*BCAS1*	Breast carcinoma amplified sequence 1	NM_003657
14.	*BMF*	Bcl2 modifying factor	NM_001003940
15.	*C1QTNF6*	C1q and tumor necrosis factor related protein 6	NM_031910
16.	*CABP2*	Calcium binding protein 2	NM_016366
17.	*CAGE1*	Cancer antigen 1	NM_001170693
18.	*CCDC81*	Coiled-coil domain containing 81	NM_021827
19.	*CCDC87*	Coiled-coil domain containing 87	NM_018219
20.	*CCL20*	Chemokine (C-C motif) ligand 20	NM_004591
21.	*CD163L1*	CD163 molecule-like 1	NM_174941
22.	*CD24*	CD24 molecule	NM_013230
23.	*CD244*	CD244 molecule, natural killer cell receptor 2B4	NM_001166663
24.	*COBL*	Cordon-bleu WH2 repeat protein	NM_015198
25.	*COL16A1*	Collagen, type XVI, alpha 1	NM_001856
26.	*CPB1*	Carboxypeptidase B1 (tissue)	NM_001871
27.	*CPXM2*	Carboxypeptidase X (M14 family), member 2	NM_198148
28.	*CSF1R*	Colony stimulating factor 1 receptor	NM_005211
29.	*CT45A1*	Cancer/testis antigen family 45, member A1	NM_001017417
30.	*CT45A5*	Cancer/testis antigen family 45, member A5	NM_001007551
31.	*CT47A11*	Cancer/testis antigen family 47, member A11	NM_173571
32.	*CTAG1A*	Cancer/testis antigen 1A	NM_139250

33.	*CTLA4*	Cytotoxic T-lymphocyte-associated protein 4	NM_005214
34.	*CYTH4*	Cytohesin 4	NM_013385
35.	*DCDC2*	Doublecortin domain containing 2	NM_016356
36.	*DHRS2*	Dehydrogenase/reductase (SDR family) member 2	NM_182908
37.	*DMBX1*	Diencephalon/mesencephalon homeobox 1	NM_147192
38.	*DNASE1L3*	Deoxyribonuclease I-like 3	NM_004944
39.	*DSCAML1*	Down syndrome cell adhesion molecule like 1	NM_020693
40.	*ECM1*	Extracellular matrix protein 1	NM_004425
41.	*EPB42*	Erythrocyte membrane protein band 4.2	NM_000119
42.	*FAM129A*	Family with sequence similarity 129, member A	NM_052966
43.	*FAM170A*	Family with sequence similarity 170, member A	NM_182761
44.	*FCN2*	Ficolin (collagen/fibrinogen domain containing lectin) 2	NM_004108
45.	*GPR17*	G protein-coupled receptor 17	BX538082
46.	*GPT*	Glutamic-pyruvate transaminase (alanine aminotransferase)	NM_005309
47.	*GRID1*	Glutamate receptor, ionotropic, delta 1	NM_017551
48.	*HEATR4*	HEAT repeat containing 4	NM_203309
49.	*HES2*	Hes family bhlh transcription factor 2	NM_019089
50.	*HES7*	Hes family bhlh transcription factor 7	NM_001165967
51.	*HKDC1*	Hexokinase domain containing 1	NM_025130
52.	*HTR1A*	5-hydroxytryptamine (serotonin) receptor 1A, G protein-coupled	NM_000524
53.	*HYAL1*	Hyaluronoglucosaminidase 1	NM_153281
54.	*IL6R*	Interleukin 6 receptor	NM_000565
55.	*KATNAL2*	Katanin p60 subunit A-like 2	NM_031303
56.	*KCNMB2*	Potassium large conductance calcium-activated channel, subfamily M, beta member 2	NM_181361
57.	*KDR*	Kinase insert domain receptor (a type III receptor tyrosine kinase)	NM_002253
58.	*KIAA0825*	Kiaa0825	NM_173665
59.	*KIAA1217*	Kiaa1217	NM_019590
60.	*KIAA1244*	Kiaa1244	NM_020340
61.	*LAMP3*	Lysosomal-associated membrane protein 3	NM_014398
62.	*LAX1*	Lymphocyte transmembrane adaptor 1	NM_017773
63.	*LGI2*	Leucine-rich repeat LGI family, member 2	NM_018176
64.	*LMO1*	LIM domain only 1 (rhombotin 1)	NM_002315
65.	*LONRF3*	LON peptidase N-terminal domain and ring finger 3	ENST00000365713
66.	*LOXHD1*	Lipoxygenase homology domains 1	AK127869
67.	*LRRC1*	Leucine rich repeat containing 1	NM_018214
68.	*MPPED1*	Metallophosphoesterase domain containing 1	NM_001044370

69.	*MUC5B*	Mucin 5B, oligomeric mucus/gel-forming	NM_002458
70.	*NEURL2*	Neuralized E3 ubiquitin protein ligase 2	NM_080749
71.	*NHS*	Nance-Horan syndrome (congenital cataracts and dental anomalies)	NM_198270
72.	*NKG7*	Natural killer cell group 7 sequence	NM_005601
73.	*NRG3*	Neuregulin 3	NM_001010848
74.	*OR1L4*	Olfactory receptor, family 1, subfamily L, member 4	NM_001005235
75.	*OR2Z1*	Olfactory receptor, family 2, subfamily Z, member 1	NM_001004699
76.	*OR52H1*	Olfactory receptor, family 52, subfamily H, member 1	NM_001005289
77.	*OR5J2*	Olfactory receptor, family 5, subfamily J, member 2	NM_001005492
78.	*OR6P1*	Olfactory receptor, family 6, subfamily P, member 1	NM_001160325
79.	*OTOP1*	Otopetrin 1	NM_177998
80.	*PAG1*	Phosphoprotein associated with glycosphingolipid microdomains 1	NM_018440
81.	*PAGE1*	P antigen family, member 1 (prostate associated)	NM_003785
82.	*PAPPA*	Pregnancy-associated plasma protein A, pappalysin 1	NM_002581
83.	*PDE4DIP*	Phosphodiesterase 4D interacting protein	NM_001198834
84.	*PDE9A*	Phosphodiesterase 9A	NM_002606
85.	*PDLIM5*	PDZ and LIM domain 5	NM_001011515
86.	*PITPNM3*	PITPNM family member 3	NM_031220
87.	*PLA2G4A*	Phospholipase A2, group IVA (cytosolic, calcium-dependent)	NM_024420
88.	*PLCD4*	Phospholipase C, delta 4	AY512961
89.	*PLD5*	Phospholipase D family, member 5	NM_152666
90.	*PLEKHB1*	Pleckstrin homology domain containing, family B (evectins) member 1	NM_021200
91.	*PLXDC2*	Plexin domain containing 2	NM_032812
92.	*PROP1*	PROP paired-like homeobox 1	NM_006261
93.	*PSG1*	Pregnancy specific beta-1-glycoprotein 1	NM_006905
94.	*PSG3*	Pregnancy specific beta-1-glycoprotein 3	NM_021016
95.	*PSG6*	Pregnancy specific beta-1-glycoprotein 6	NM_002782
96.	*PSG8*	Pregnancy specific beta-1-glycoprotein 8	NM_182707
97.	*PSG9*	Pregnancy specific beta-1-glycoprotein 9	NM_002784
98.	*RNF180*	Ring finger protein 180	NM_178532
99.	*RNPC3*	RNA-binding region (RNP1, RRM) containing 3	XM_005271009
100.	*S100A16*	S100 calcium binding protein A16	NM_080388
101.	*S1PR4*	Sphingosine-1-phosphate receptor 4	NM_003775
102.	*SH2D1A*	SH2 domain containing 1A	NM_001114937
103.	*SHBG*	Sex hormone-binding globulin	NM_001040
104.	*SIAH3*	Siah E3 ubiquitin protein ligase family member 3	NM_198849

105.	*SIM2*	Single-minded family bhlh transcription factor 2	NM_009586
106.	*SLC24A3*	Solute carrier family 24 (sodium/potassium/calcium exchanger), member 3	NM_020689
107.	*SNAI3*	Snail family zinc finger 3	NM_178310
108.	*SORL1*	Sortilin-related receptor, L(DLR class) A repeats containing	NM_003105
109.	*SPATA31D3*	SPATA31 subfamily D, member 3	NM_207416
110.	*SSX4B*	Synovial sarcoma, X breakpoint 4B	NM_001034832
111.	*STAG3*	Stromal antigen 3	NM_001282717
112.	*STARD13*	Star-related lipid transfer (START) domain containing 13	NM_178006
113.	*SYNE1*	Spectrin repeat containing, nuclear envelope 1	AL713682
114.	*SYT8*	Synaptotagmin VIII	NM_138567
115.	*TDO2*	Tryptophan 2,3-dioxygenase	NM_005651
116.	*TEC*	Tec protein tyrosine kinase	NM_003215
117.	*TFAP2E*	Transcription factor AP-2 epsilon (activating enhancer binding protein 2 epsilon)	NM_178548
118.	*TGFBI*	Transforming growth factor, beta-induced, 68kda	NM_000358
119.	*TIPARP*	TCDD-inducible poly(ADP-ribose) polymerase	NM_001184717
120.	*TM4SF19*	Transmembrane 4 L six family member 19	NM_138461
121.	*TMEM106A*	Transmembrane protein 106A	NM_145041
122.	*TMEM131*	Transmembrane protein 131	NM_015348
123.	*TP63*	Tumor protein p63	NM_003722
124.	*TTC39A*	Tetratricopeptide repeat domain 39A	NM_001080494
125.	*UGT2B11*	UDP glucuronosyltransferase 2 family, polypeptide B11	NM_001073
126.	*ULK2*	Unc-51 like autophagy activating kinase 2	NM_014683
127.	*UPP1*	Uridine phosphorylase 1	NM_181597
128.	*UQCRH*	Ubiquinol-cytochrome c reductase hinge protein	NM_006004
129.	*VIP*	Vasoactive intestinal peptide	NM_003381
130.	*VIPR2*	Vasoactive intestinal peptide receptor 2	NM_003382
131.	*WDR20*	WD repeat domain 20	NM_001242415
132.	*WNT5A*	Wingless-type MMTV integration site family, member 5A	NM_003392
133.	*ZG16B*	Zymogen granule protein 16B	NM_145252
134.	*ZNF385D*	Zinc finger protein 385D	ENST00000494108
135.	*ZNRF2*	Zinc and ring finger 2	TCONS_00012988

Table S3. *Oncogenic Profile of gip2-depenndent Genes*

	GENE	Oncogenic Role	References
1.	*ABLIM1*	Cell migration in hepatocellular carcinoma	Dong *et al*., 2020 [1]
2.	*ACKR3*	Multiple oncogenic pathways in breast, lung, and brain cancer	Neves *et al*., 2019 [2]
3.	*AHNAK2*	Oncogenic and prognostic marker in clear cell renal cell carcinoma and pancreatic cancer	Wang *et al*., 2017; Lu *et al*., 2017 [3,4]
4.	*APBA1*	Pro-survival gene in cervical cancer cells	Guo *et al*., 2019 [5]
5.	*APOC1*	Oncogenic metastasis in clear cell renal cell carcinoma	Li *et al*., 2020 [6]
6.	*ARHGEF28*	Cell Motility and invasion in Colon carcinoma	Yu *et al*., 2011 [7]
7.	*ASS1*	Gastric cancer invasion	Tsai *et al*., 2018 [8]
8.	*ATAT1*	Proliferation and invasion colon cancer cells	Oh *et al*., 2017 [9]
9.	*BAD*	Marker of triple negative and poor outcome in breast cancer	Boac *et al*., 2019 [10]
10.	*C1QTNF6*	Oncogenic in non-small cell lung carcinoma cells	Zhang and Feng, 2021 [11]
11.	*CAGE1*	Biomarker in different cancers	Park *et al*., 2003 [12]
12.	*CCDC81*	Biomarker in nasopharyngeal and small cell lung cancers	Zhang *et al*., 2019 Iwakawa *et al*., 2013 [13,14]
13.	*CCL20*	Promotes accelerated cancer growth in multiple cancers	Kadamoto *et al*., 2020 [15]
14.	*CD24*	Cancer progression & Metastasis in multiple cancers	Duex *et al*., 2017 [16]
15.	*CD244*	Therapeutic target in head and neck cancer	Agresta *et al*., 2020 [17]
16.	*COL16A1*	Oncogenic proliferation in oral cancer	Ratzinger *et al*., 2011 [18]
17.	*CPB1*	Pancreatic cancer susceptibility gene,	Tamura *et al*., 2018 [19]
18.	*CPXM2*	Proliferation and migration in gastric cancer cells; poor prognosis marker	Niu *et al*., 2019 [20]
19.	*CSF1R*	Oncogenic in T cell lymphoma; proliferation and migration in ovarian cancer cells	Chambers *et al*., 2010 [21,22]
20.	*CT45A1*	Protooncogene in breast cancer	Gao *et al*., 2014 [23]
21.	*CTLA4*	Poor prognosis indicator in multiple cancers	Santoni *et al*., 2018; Zhao *et al*., 2018 [24,25]
22.	*CYTH4*	Overexpression in ovarian cancers	Zhang *et al*., 2020 [26]
23.	*DCDC2*	Oncogenic migration in prostate cancer	Longoni *et al*., 2013 [27]
24.	*DMBX1*	Oncogenic proliferation in many cancers	Luo *et al*., 2019 [28]
25.	*ECM1*	Metabolic reprogramming in gastric cancer	Meng *et al*., 2018 [29]
26.	*FAM129A*	Proliferation and invasive migration in non-small cell lung carcinoma cells	Zhang *et al*., 2019 [30]

27.	*GPT1*	Hepatocellular carcinoma growth and progression	Guo *et al*., 2020 [31]
28.	*GRID1*	Negatively correlated with overall survival of endometrial cancer patients,	Wang *et al*., 2020 [32]
29.	*HES2*	Associated with colorectal cancer,	Katoh & Katoh, 2007 [33]
30.	*HES7*	Cancer stemness in many cancers, EMT:	Song *et al*., 2019 [34]
31.	*HKDC1*	Tumorigenesis & Glycolytic pathway lung adenocarcinoma,	Wang *et al*., 2020 [35]
32.	*HTR1A*	Expression in breast cancer cells,	Kopparapu eta al., 2013 [36]
33.	*HYAL1*	Tumor cell proliferation, migration, invasion, and angiogenesis in breast cancer	Tan *et al*., 2011 [37]
34.	*IL6R*	Tumor promotion in multiple cancers; EMT in colorectal cancers	Rokavec *et al*., 2014 [38]
35.	*KDR*	Proliferation and cancer cell metabolism in ovarian cancer,	Cybulski *et al*., 2012; Chen *et al*., 2019 [39,40]
36.	*LAMP3*	Associated with poor prognosis in ovarian cancer	Wang *et al*., 2017 [41]
37.	*LAX1*	Identified as shorter DFS marker in ovarian cancer	Yin *et al*., 2019 [42]
38.	*LMO1*	Tumorigenic in multiple cancers	Zhao *et al*., 2021 [43]
39.	*LOXHD1*	Associated with breast cancer growth and progression,	Schulten *et al*., 2017 [44]
40.	*LRRC1*	Oncogenic role in hepatocellular carcinoma	Li *et al*., 2013 [45]
41.	*MUC5B*	Tumor promotion in multiple cancers including gastrointestinal cancers	Lahdaoui *et al*., 2017 [46]
42.	*NEURL2*	Stemness in lung cancer cells,	Liu *et al*., 2019 [47]
43.	*NRG3*	Oncogenic signaling pathways in many cancers	Montero *et al*., 2008 [48]
44.	*PAG1*	Confers radio-resistance laryngeal cancer	Shen *et al*., 2018 [49]
45.	*PAGE1*	Tumor antigen in many different cancers	Chen *et al*., 1998 [50]
46.	*PAPPA*	Oncogenic in many cancers	Guo *et al*., 2018 [51]
47.	*PDE4DIP*	Oncogenic in pineoblastoma	Snuderl *et al*., 2018 [52]
48.	*PDE9A*	Expression is correlated with malignant breast cancer	Karami-Tehrani *et al*., 2012 [53]
49.	*PDLIM5*	Migration and Invasion in lung cancer cells,	Shi *et al*., 2020 [54]
50.	*PITPNM3*	Breast cancer metastasis	Chen *et al*., 2011 [55]
51.	*PLA2G4A*	Associated with poor prognosis in many cancers including acute myeloid leukemia	Bai *et al*., 2020 [56]
52.	*PLCD4*	Associated with increased proliferation of breast cancer cells	Leung *et al*., 2004 [57]
53.	*PLXDC2*	Associated with paclitaxel resistance in ovarian cancer	Wang *et al*., 2018 [58]
54.	*PROP1*	Persistent expression is associated with pituitary tumors	Cushman *et al*., 2001 [59]
55.	*PSG1*	Associated with chemoresistance in breast cancer	He *et al*., 2016 [60]
56.	*PSG6*	Associated with increased mortality in Stomach Adenocarcinoma	Wang *et al*., 2020 [61]

57.	*PSG9*	Accelerated growth and progression in many cancers including breast cancer,	Yang *et al*., 2016 [62]
58.	*S100A16*	Promotes Metastasis in pancreatic adeno ductal carcinoma cells	Fang *et al*., 2021 [63]
59.	*S1PR4*	Invasion and metastasis of prostate cancer cells,	Lee *et al*., 2019 [64]
60.	*SH2D1A*	Associated with metastasis in breast cancer,	Park *et al*., 2020 [65]
61.	*SHBG*	Associated with poor prognosis in ovarian cancer	Huang *et al*., 2013 [66]
62.	*SNAI3*	Tumor progression in Colorectal cancer	Chen *et al*., 2018 [67]
63.	*SORL1*	Contributes to therapy resistance in breast cancer	Al-Akhrass *et al*., 2021 [68]
64.	*SYNE1*	Hepatocellular carcinoma growth and progression,	Faraj Shaglouf *et al*., 2020 [69]
65.	*SYT8*	Peritoneal Metastasis of gastric Cancer	Kanda *et al*., 2018 [70]
66.	*TDO2*	Proliferation, migration, and invasion of ovarian cancer cells	Zhao *et al*., 2021 [71]
67.	*TEC*	Oncogenic role in in many cancers including hepatocellular carcinoma	Vanova *et al*., 2017 [72]
68.	*TFAP2E*	Associated with chemoresistance colorectal cancer	Ebert *et al*., 2012 [73]
69.	*TGFBI*	Therapy resistance in breast cancer	Palomeras *et al*., 2019; Huynh *et al*, 2019 [74,75]
70.	*TP63*	Invasive phenotype in basal carcinoma	Palmbos *et al*., 2019 [76]
71.	*UPP1*	Potential role in thyroid carcinoma epithelial-mesenchymal transition	Guan *et al*., 2019 [77]
72.	*UQCRH*	Indicator of poor prognosis in hepatocellular carcinoma	Park *et al*., 2017 [78]
73.	*VIP*	Oncogenic autocrine factor in many cancers including lung cancer	Moody *et al*., 2000 [79]
74.	*VIPR2*	Oncogenic signaling pathways in many cancers,	Moody *et al*., 2016 [80]
75.	*WDR20*	Promotes cancer cell survival in multiple cancers including and prostate cancer	McClurg *et al*., 2015 [81]
76.	*WNT5A*	Cancer cell invasion, metastasis, metabolism, and inflammation in many cancers,	Asem *et al*., 2016 [82]
77.	*ZG16B*	Oncogenic in many cancers; cell cycle progression via Wnt/b-CNN pathway in colorectal cancer	Escudero-Paniagua *et al*., 2020 [83]
78.	*ZNRF2*	Stimulates cell proliferation along with the suppression of apoptosis in non-small cell lung carcinoma cells	Zhang *et al*., 2016 [84]

Cited References

1 Dong, X. *et al.* Rictor promotes cell migration and actin polymerization through regulating ABLIM1 phosphorylation in Hepatocellular Carcinoma. *Int J Biol Sci* **16**, 2835-2852, doi:10.7150/ijbs.46285 (2020).

2 Neves, M. *et al.* The Role of ACKR3 in Breast, Lung, and Brain Cancer. *Mol Pharmacol* **96**, 819-825, doi:10.1124/mol.118.115279 (2019).

3 Wang, M. *et al.* AHNAK2 is a Novel Prognostic Marker and Oncogenic Protein for Clear Cell Renal Cell Carcinoma. *Theranostics* **7**, 1100-1113, doi:10.7150/thno.18198 (2017).

4 Lu, D., Wang, J., Shi, X., Yue, B. & Hao, J. AHNAK2 is a potential prognostic biomarker in patients with PDAC. *Oncotarget* **8**, 31775-31784, doi:10.18632/oncotarget.15990 (2017).

5 Guo, W. *et al.* Effect of hyperoside on cervical cancer cells and transcriptome analysis of differentially expressed genes. *Cancer Cell Int* **19**, 235, doi:10.1186/s12935-019-0953-4 (2019).

6 Li, Y. L. *et al.* ApoC1 promotes the metastasis of clear cell renal cell carcinoma via activation of STAT3. *Oncogene* **39**, 6203-6217, doi:10.1038/s41388-020-01428-3 (2020).

7 Yu, H. G. *et al.* p190RhoGEF (Rgnef) promotes colon carcinoma tumor progression via interaction with focal adhesion kinase. *Cancer Res* **71**, 360-370, doi:10.1158/0008-5472.CAN-10-2894 (2011).

8 Tsai, C. Y. *et al.* Argininosuccinate synthetase 1 contributes to gastric cancer invasion and progression by modulating autophagy. *FASEB J* **32**, 2601-2614, doi:10.1096/fj.201700094R (2018).

9 Oh, S. *et al.* Genetic disruption of tubulin acetyltransferase, alphaTAT1, inhibits proliferation and invasion of colon cancer cells through decreases in Wnt1/beta-catenin signaling. *Biochem Biophys Res Commun* **482**, 8-14, doi:10.1016/j.bbrc.2016.11.039 (2017).

10 Boac, B. M. *et al.* Expression of the BAD pathway is a marker of triple-negative status and poor outcome. *Sci Rep* **9**, 17496, doi:10.1038/s41598-019-53695-0 (2019).

11 Zhang, W. & Feng, G. C1QTNF6 regulates cell proliferation and apoptosis of NSCLC in vitro and in vivo. *Biosci Rep* **41**, doi:10.1042/BSR20201541 (2021).

12 Park, S. *et al.* Identification and characterization of a novel cancer/testis antigen gene CAGE-1. *Biochim Biophys Acta* **1625**, 173-182, doi:10.1016/s0167-4781(02)00620-6 (2003).

13 Zhang, J. Z., Wu, Z. H. & Cheng, Q. Screening and identification of key biomarkers in nasopharyngeal carcinoma: Evidence from bioinformatic analysis. *Medicine (Baltimore)* **98**, e17997, doi:10.1097/MD.0000000000017997 (2019).

14 Iwakawa, R. *et al.* Genome-wide identification of genes with amplification and/or fusion in small cell lung cancer. *Genes Chromosomes Cancer* **52**, 802-816, doi:10.1002/gcc.22076 (2013).

15 Kadomoto, S., Izumi, K. & Mizokami, A. The CCL20-CCR6 Axis in Cancer Progression. *Int J Mol Sci* **21**, doi:10.3390/ijms21155186 (2020).

16 Duex, J. E. *et al.* Nuclear CD24 Drives Tumor Growth and Is Predictive of Poor Patient Prognosis. *Cancer Res* **77**, 4858-4867, doi:10.1158/0008-5472.CAN-17-0367 (2017).

17 Agresta, L. *et al.* CD244 represents a new therapeutic target in head and neck squamous cell carcinoma. *J Immunother Cancer* **8**, doi:10.1136/jitc-2019-000245 (2020).

18 Ratzinger, S., Grassel, S., Dowejko, A., Reichert, T. E. & Bauer, R. J. Induction of type XVI collagen expression facilitates proliferation of oral cancer cells. *Matrix Biol* **30**, 118-125, doi:10.1016/j.matbio.2011.01.001 (2011).

19 Tamura, K. *et al.* Mutations in the pancreatic secretory enzymes CPA1 and CPB1 are associated with pancreatic cancer. *Proc Natl Acad Sci U S A* **115**, 4767-4772, doi:10.1073/pnas.1720588115 (2018).

20 Niu, G. *et al.* Overexpression of CPXM2 predicts an unfavorable prognosis and promotes the proliferation and migration of gastric cancer. *Oncol Rep* **42**, 1283-1294, doi:10.3892/or.2019.7254 (2019).

21 Chambers, S. K. Role of CSF-1 in progression of epithelial ovarian cancer. *Future Oncol* **5**, 1429-1440, doi:10.2217/fon.09.103 (2009).

22 Murga-Zamalloa, C. *et al.* Colony-Stimulating Factor 1 Receptor (CSF1R) Activates AKT/mTOR Signaling and Promotes T-Cell Lymphoma Viability. *Clin Cancer Res* **26**, 690-703, doi:10.1158/1078-0432.CCR-19-1486 (2020).

23 Shang, B. *et al.* CT45A1 acts as a new proto-oncogene to trigger tumorigenesis and cancer metastasis. *Cell death & disease* **5**, e1285, doi:10.1038/cddis.2014.244 (2014).

24 Santoni, G. *et al.* High CTLA-4 expression correlates with poor prognosis in thymoma patients. *Oncotarget* **9**, 16665-16677, doi:10.18632/oncotarget.24645 (2018).

25 Zhao, Y. *et al.* Evolving Roles for Targeting CTLA-4 in Cancer Immunotherapy. *Cell Physiol Biochem* **47**, 721-734, doi:10.1159/000490025 (2018).

26 Zhang, Q., Wang, Q., Wu, S. & Zhang, J. Clinical implication and immunological characterisation of the ARF-GEF family member CYTH4 in ovarian cancer. *Autoimmunity* **53**, 434-442, doi:10.1080/08916934.2020.1836487 (2020).

27 Longoni, N. *et al.* Aberrant expression of the neuronal-specific protein DCDC2 promotes malignant phenotypes and is associated with prostate cancer progression. *Oncogene* **32**, 2315-2324, 2324 e2311-2314, doi:10.1038/onc.2012.245 (2013).

28 Luo, J. *et al.* DMBX1 promotes tumor proliferation and regulates cell cycle progression via repressing OTX2-mediated transcription of p21 in lung adenocarcinoma cell. *Cancer Lett* **453**, 45-56, doi:10.1016/j.canlet.2019.03.045 (2019).

29 Gan, L. *et al.* Extracellular matrix protein 1 promotes cell metastasis and glucose metabolism by inducing integrin beta4/FAK/SOX2/HIF-1alpha signaling pathway in gastric cancer. *Oncogene* **37**, 744-755, doi:10.1038/onc.2017.363 (2018).

30 Zhang, N. *et al.* FAM129A promotes invasion and proliferation by activating FAK signaling pathway in non-small cell lung cancer. *Int J Clin Exp Pathol* **12**, 893-900 (2019).

31 Guo, W., Tan, H. Y., Li, S., Wang, N. & Feng, Y. Glutamic-Pyruvic Transaminase 1 Facilitates Alternative Fuels for Hepatocellular Carcinoma Growth-A Small Molecule Inhibitor, Berberine. *Cancers (Basel)* **12**, doi:10.3390/cancers12071854 (2020).

32 Wang, H. *et al.* Prognostic value of an autophagy-related gene expression signature for endometrial cancer patients. *Cancer Cell Int* **20**, 306, doi:10.1186/s12935-020-01413-6 (2020).

33 Katoh, M. & Katoh, M. Identification and characterization of human HES2, HES3, and HES5 genes in silico. *Int J Oncol* **25**, 529-534 (2004).

34 Song, J. *et al.* Epithelial-mesenchymal transition markers screened in a cell-based model and validated in lung adenocarcinoma. *BMC Cancer* **19**, 680, doi:10.1186/s12885-019-5885-9 (2019).

35 Wang, X. *et al.* HKDC1 promotes the tumorigenesis and glycolysis in lung adenocarcinoma via regulating AMPK/mTOR signaling pathway. *Cancer Cell Int* **20**, 450, doi:10.1186/s12935-020-01539-7 (2020).

36 Kopparapu, P. K., Tinzl, M., Anagnostaki, L., Persson, J. L. & Dizeyi, N. Expression and localization of serotonin receptors in human breast cancer. *Anticancer Res* **33**, 363-370 (2013).

37 Tan, J. X. *et al.* Upregulation of HYAL1 expression in breast cancer promoted tumor cell proliferation, migration, invasion and angiogenesis. *PLoS One* **6**, e22836, doi:10.1371/journal.pone.0022836 (2011).

38 Rokavec, M. *et al.* IL-6R/STAT3/miR-34a feedback loop promotes EMT-mediated colorectal cancer invasion and metastasis. *J Clin Invest* **124**, 1853-1867, doi:10.1172/JCI73531 (2014).

39 Cybulski, M. *et al.* Cyclin I correlates with VEGFR-2 and cell proliferation in human epithelial ovarian cancer. *Gynecol Oncol* **127**, 217-222, doi:10.1016/j.ygyno.2012.06.038 (2012).
40 Chen, L. *et al.* Apatinib inhibits glycolysis by suppressing the VEGFR2/AKT1/SOX5/GLUT4 signaling pathway in ovarian cancer cells. *Cell Oncol (Dordr)* **42**, 679-690, doi:10.1007/s13402-019-00455-x (2019).
41 Wang, D. *et al.* LAMP3 expression correlated with poor clinical outcome in human ovarian cancer. *Tumour Biol* **39**, 1010428317695014, doi:10.1177/1010428317695014 (2017).
42 Yin, F. *et al.* Microarray-based identification of genes associated with prognosis and drug resistance in ovarian cancer. *J Cell Biochem* **120**, 6057-6070, doi:10.1002/jcb.27892 (2019).
43 Zhao, G. F., Du, L. Q., Zhang, L. & Jia, Y. C. LIM domain only 1: an oncogenic transcription cofactor contributing to the tumorigenesis of multiple cancer types. *Chin Med J (Engl)* **134**, 1017-1030, doi:10.1097/CM9.0000000000001487 (2021).
44 Schulten, H. J. *et al.* Comprehensive molecular biomarker identification in breast cancer brain metastases. *J Transl Med* **15**, 269, doi:10.1186/s12967-017-1370-x (2017).
45 Li, Y., Zhou, B., Dai, J., Liu, R. & Han, Z. G. Aberrant upregulation of LRRC1 contributes to human hepatocellular carcinoma. *Mol Biol Rep* **40**, 4543-4551, doi:10.1007/s11033-013-2549-8 (2013).
46 Lahdaoui, F. *et al.* Depletion of MUC5B mucin in gastrointestinal cancer cells alters their tumorigenic properties: implication of the Wnt/beta-catenin pathway. *Biochem J* **474**, 3733-3746, doi:10.1042/BCJ20170348 (2017).
47 Liu, D. *et al.* Asymmetric Division Gene Neurl2 Mediates Twist2 Regulation of Self-Renewal of Mouse Lewis Lung Cancer Stem Cells. *J Cancer* **10**, 3381-3388, doi:10.7150/jca.31553 (2019).
48 Montero, J. C. *et al.* Neuregulins and cancer. *Clin Cancer Res* **14**, 3237-3241, doi:10.1158/1078-0432.CCR-07-5133 (2008).
49 Shen, L. *et al.* PAG1 promotes the inherent radioresistance of laryngeal cancer cells via activation of STAT3. *Exp Cell Res* **370**, 127-136, doi:10.1016/j.yexcr.2018.06.014 (2018).
50 Chen, M. E., Lin, S. H., Chung, L. W. & Sikes, R. A. Isolation and characterization of PAGE-1 and GAGE-7. New genes expressed in the LNCaP prostate cancer progression model that share homology with melanoma-associated antigens. *The Journal of biological chemistry* **273**, 17618-17625, doi:10.1074/jbc.273.28.17618 (1998).
51 Guo, Y., Bao, Y., Guo, D. & Yang, W. Pregnancy-associated plasma protein a in cancer: expression, oncogenic functions and regulation. *Am J Cancer Res* **8**, 955-963 (2018).
52 Snuderl, M. *et al.* Recurrent homozygous deletion of DROSHA and microduplication of PDE4DIP in pineoblastoma. *Nat Commun* **9**, 2868, doi:10.1038/s41467-018-05029-3 (2018).
53 Karami-Tehrani, F., Moeinifard, M., Aghaei, M. & Atri, M. Evaluation of PDE5 and PDE9 expression in benign and malignant breast tumors. *Arch Med Res* **43**, 470-475, doi:10.1016/j.arcmed.2012.08.006 (2012).
54 Shi, Y. *et al.* PDLIM5 inhibits STUB1-mediated degradation of SMAD3 and promotes the migration and invasion of lung cancer cells. *The Journal of biological chemistry* **295**, 13798-13811, doi:10.1074/jbc.RA120.014976 (2020).
55 Chen, J. *et al.* CCL18 from tumor-associated macrophages promotes breast cancer metastasis via PITPNM3. *Cancer Cell* **19**, 541-555, doi:10.1016/j.ccr.2011.02.006 (2011).
56 Bai, H., Zhou, M., Zeng, M. & Han, L. PLA2G4A Is a Potential Biomarker Predicting Shorter Overall Survival in Patients with Non-M3/NPM1 Wildtype Acute Myeloid Leukemia. *DNA Cell Biol* **39**, 700-708, doi:10.1089/dna.2019.5187 (2020).
57 Leung, D. W. *et al.* Phospholipase C delta-4 overexpression upregulates ErbB1/2 expression, Erk signaling pathway, and proliferation in MCF-7 cells. *Mol Cancer* **3**, 15, doi:10.1186/1476-4598-3-15 (2004).

58 Wang, Y. & Li, H. Identification of proteins associated with paclitaxel resistance of epithelial ovarian cancer using iTRAQ-based proteomics. *Oncol Lett* **15**, 9793-9801, doi:10.3892/ol.2018.8600 (2018).
59 Cushman, L. J. *et al.* Persistent Prop1 expression delays gonadotrope differentiation and enhances pituitary tumor susceptibility. *Hum Mol Genet* **10**, 1141-1153, doi:10.1093/hmg/10.11.1141 (2001).
60 He, D. X. *et al.* Targeting PSG1 to enhance chemotherapeutic efficacy: new application for anti-coagulant the dicumarol. *Clin Sci (Lond)* **130**, 2267-2276, doi:10.1042/CS20160536 (2016).
61 Wang, H., Shen, L., Li, Y. & Lv, J. Integrated characterisation of cancer genes identifies key molecular biomarkers in stomach adenocarcinoma. *J Clin Pathol* **73**, 579-586, doi:10.1136/jclinpath-2019-206400 (2020).
62 Yang, L. *et al.* Pregnancy-specific glycoprotein 9 (PSG9), a driver for colorectal cancer, enhances angiogenesis via activation of SMAD4. *Oncotarget* **7**, 61562-61574, doi:10.18632/oncotarget.11146 (2016).
63 Fang, D. *et al.* S100A16 promotes metastasis and progression of pancreatic cancer through FGF19-mediated AKT and ERK1/2 pathways. *Cell Biol Toxicol*, doi:10.1007/s10565-020-09574-w (2021).
64 Lee, C. F. *et al.* Activation of sphingosine kinase by lipopolysaccharide promotes prostate cancer cell invasion and metastasis via SphK1/S1PR4/matriptase. *Oncogene* **38**, 5580-5598, doi:10.1038/s41388-019-0833-3 (2019).
65 Park, S. B., Hwang, K. T., Chung, C. K., Roy, D. & Yoo, C. Causal Bayesian gene networks associated with bone, brain and lung metastasis of breast cancer. *Clin Exp Metastasis* **37**, 657-674, doi:10.1007/s10585-020-10060-0 (2020).
66 Huang, R. *et al.* Sex hormone-binding globulin (SHBG) expression in ovarian carcinomas and its clinicopathological associations. *PLoS One* **8**, e83238, doi:10.1371/journal.pone.0083238 (2013).
67 Chen, C. *et al.* FOXD4 induces tumor progression in colorectal cancer by regulation of the SNAI3/CDH1 axis. *Cancer biology & therapy* **19**, 1065-1071, doi:10.1080/15384047.2018.1480291 (2018).
68 Al-Akhrass, H. *et al.* A feed-forward loop between SorLA and HER3 determines heregulin response and neratinib resistance. *Oncogene* **40**, 1300-1317, doi:10.1038/s41388-020-01604-5 (2021).
69 Faraj Shaglouf, L. H., Ranjpour, M., Wajid, S. & Jain, S. K. Elevated expression of cellular SYNE1, MMP10, and GTPase1 and their regulatory role in hepatocellular carcinoma progression. *Protoplasma* **257**, 157-167, doi:10.1007/s00709-019-01423-w (2020).
70 Kanda, M. *et al.* Significance of SYT8 For the Detection, Prediction, and Treatment of Peritoneal Metastasis From Gastric Cancer. *Ann Surg* **267**, 495-503, doi:10.1097/SLA.0000000000002096 (2018).
71 Zhao, Y. *et al.* Tryptophan 2, 3dioxygenase promotes proliferation, migration and invasion of ovarian cancer cells. *Mol Med Rep* **23**, doi:10.3892/mmr.2021.12084 (2021).
72 Vanova, T. *et al.* Tyrosine Kinase Expressed in Hepatocellular Carcinoma, TEC, Controls Pluripotency and Early Cell Fate Decisions of Human Pluripotent Stem Cells via Regulation of Fibroblast Growth Factor-2 Secretion. *Stem Cells* **35**, 2050-2059, doi:10.1002/stem.2660 (2017).
73 Ebert, M. P. *et al.* TFAP2E-DKK4 and chemoresistance in colorectal cancer. *N Engl J Med* **366**, 44-53, doi:10.1056/NEJMoa1009473 (2012).
74 Palomeras, S. *et al.* Epigenetic silencing of TGFBI confers resistance to trastuzumab in human breast cancer. *Breast Cancer Res* **21**, 79, doi:10.1186/s13058-019-1160-x (2019).
75 Huynh, L. K., Hipolito, C. J. & Ten Dijke, P. A Perspective on the Development of TGF-beta Inhibitors for Cancer Treatment. *Biomolecules* **9**, doi:10.3390/biom9110743 (2019).
76 Palmbos, P. L. *et al.* ATDC mediates a TP63-regulated basal cancer invasive program. *Oncogene* **38**, 3340-3354, doi:10.1038/s41388-018-0646-9 (2019).

77 Guan, Y., Bhandari, A., Zhang, X. & Wang, O. Uridine phosphorylase 1 associates to biological and clinical significance in thyroid carcinoma cell lines. *J Cell Mol Med* **23**, 7438-7448, doi:10.1111/jcmm.14612 (2019).
78 Park, E. R. *et al.* The mitochondrial hinge protein, UQCRH, is a novel prognostic factor for hepatocellular carcinoma. *Cancer Med* **6**, 749-760, doi:10.1002/cam4.1042 (2017).
79 Moody, T. W., Walters, J., Casibang, M., Zia, F. & Gozes, Y. VPAC1 receptors and lung cancer. *Ann N Y Acad Sci* **921**, 26-32, doi:10.1111/j.1749-6632.2000.tb06947.x (2000).
80 Moody, T. W., Nuche-Berenguer, B. & Jensen, R. T. Vasoactive intestinal peptide/pituitary adenylate cyclase activating polypeptide, and their receptors and cancer. *Curr Opin Endocrinol Diabetes Obes* **23**, 38-47, doi:10.1097/MED.0000000000000218 (2016).
81 McClurg, U. L. *et al.* Ubiquitin-specific protease 12 interacting partners Uaf-1 and WDR20 are potential therapeutic targets in prostate cancer. *Oncotarget* **6**, 37724-37736, doi:10.18632/oncotarget.6075 (2015).
82 Asem, M. S., Buechler, S., Wates, R. B., Miller, D. L. & Stack, M. S. Wnt5a Signaling in Cancer. *Cancers (Basel)* **8**, doi:10.3390/cancers8090079 (2016).
83 Escudero-Paniagua, B. *et al.* PAUF/ZG16B promotes colorectal cancer progression through alterations of the mitotic functions and the Wnt/beta-catenin pathway. *Carcinogenesis* **41**, 203-213, doi:10.1093/carcin/bgz093 (2020).
84 Zhang, X. F. *et al.* The role of ZNRF2 in the growth of non-small cell lung cancer. *Eur Rev Med Pharmacol Sci* **20**, 4011-4017 (2016).

Table S4. ***Network Gene Alteration Frequency in Ovarian Cancer Patients***

Expression profiles of the growth-promoting oncogenic genes identified by datamining were analyzed for their expression profile using TCGA ovarian serous cystadenocarcinoma cancer dataset (TCGA Firehose Legacy). Number patients in which the expression of the respective genes were altered and the % patients altered (> 5%) were extracted from CBioPortal.

	**Gene**	**Patients Altered**	**Percent Patients Altered**
1	*GPT*	103	33%
2	*LAMP3*	91	29%
3	*TP63*	81	26%
4	*CPB1*	63	20%
5	*ECM1*	60	19%
6	*CCDC81*	49	16%
7	*DMBX1*	44	14%
8	*CAGE1*	43	14%
9	*LAX1*	43	14%
10	*VIPR2*	43	14%
11	*PDE4DIP*	41	13%
12	*NIBAN1*	40	13%
13	*PAG1*	38	12%
14	*CTLA4*	36	12%
15	*UQCRH*	36	12%
16	*COL16A1*	34	11%
17	*LOXHD1*	31	10%
18	*S100A16*	31	10%
19	*PAGE1*	30	10%
20	*TFAP2E*	30	10%
21	*DCDC2*	29	9%
22	*LMO1*	29	9%
23	*SORL1*	29	9%
24	*CPXM2*	28	9%
25	*PROP1*	28	9%
26	*SYT8*	28	9%
27	*IL6R*	27	9%
28	*ACKR3*	26	8%
29	*C1QTNF6*	26	8%
30	*PLA2G4A*	26	8%
31	*SYNE1*	26	8%
32	*CCL20*	25	8%
33	*KDR*	25	8%
34	*TGFBI*	25	8%
35	*CD244*	25	8%
36	*NRG3*	24	8%
37	*PSG1*	24	8%
38	*SNAI3*	24	8%
39	*BAD*	24	7%
40	*APOC1*	23	7%
41	*MUC5B*	22	7%
42	*TDO2*	22	7%
43	*ATAT1*	22	7%
44	*PLXDC2*	21	7%
45	*LRRC1*	21	6%

46	*WNT5A*	20	6%
47	*PSG6*	20	6%
48	*ABLIM1*	19	6%
49	*PDLIM5*	18	6%
50	*APBA1*	18	5%
51	*HES2*	17	5%
52	*NEURL2*	17	5%
53	*WDR20*	17	5%
54	*AHNAK2*	16	5%
55	*CSF1R*	16	5%
56	*PITPNM3*	16	5%
57	*PSG9*	16	5%
58	*UPP1*	16	5%
59	*ASS1*	14	5%
60	*PLCD4*	14	5%
61	*VIP*	14	5%

Table S5. ***Co-occurrence of Network Genes in Ovarian Cancer Patients***

Network genes that show increased expression in more than 5% of the genes were analyzed for their co-expression using the co-occurrence parameter of the CBioPortal. Co-occurrence profile of the genes with the p-value of <0.001, was extracted from the CBioPortal using Ovarian Serous Cystadenocarcinoma (TCGA, Firehose Legacy). The dataset available at https://www.cbioportal.org.

Gene	*Gene*	*p-Value*	*Tendency*
LAMP3	*TP63*	<0.001	Co-occurrence
S100A16	*IL6R*	<0.001	Co-occurrence
PSG6	*PSG9*	<0.001	Co-occurrence
PSG1	*PSG6*	<0.001	Co-occurrence
PSG1	*PSG9*	<0.001	Co-occurrence
NIBAN1	*PLA2G4A*	<0.001	Co-occurrence
DMBX1	*UQCRH*	<0.001	Co-occurrence
ECM1	*S100A16*	<0.001	Co-occurrence
DCDC2	*ATAT1*	<0.001	Co-occurrence
LAMP3	*CPB1*	<0.001	Co-occurrence
ECM1	*IL6R*	<0.001	Co-occurrence
TP63	*CPB1*	<0.001	Co-occurrence
APOC1	*PSG9*	<0.001	Co-occurrence
IL6R	*CD244*	<0.001	Co-occurrence
CCDC81	*BAD*	<0.001	Co-occurrence
COL16A1	*TGFBI*	<0.001	Co-occurrence
APOC1	*PSG6*	<0.001	Co-occurrence
NIBAN1	*CD244*	<0.001	Co-occurrence
ECM1	*PDE4DIP*	<0.001	Co-occurrence
KDR	*PDLIM5*	<0.001	Co-occurrence
PSG1	*APOC1*	<0.001	Co-occurrence
COL16A1	*C1QTNF6*	<0.001	Co-occurrence
ACKR3	*PLCD4*	<0.001	Co-occurrence
S100A16	*CD244*	<0.001	Co-occurrence
PLA2G4A	*CD244*	<0.001	Co-occurrence
CAGE1	*DCDC2*	<0.001	Co-occurrence
PDE4DIP	*S100A16*	<0.001	Co-occurrence
CAGE1	*ATAT1*	<0.001	Co-occurrence
UQCRH	*TFAP2E*	<0.001	Co-occurrence
PDE4DIP	*IL6R*	<0.001	Co-occurrence
CTLA4	*DCDC2*	<0.001	Co-occurrence
PAG1	*NRG3*	<0.001	Co-occurrence
LAX1	*NIBAN1*	<0.001	Co-occurrence
C1QTNF6	*TGFBI*	<0.001	Co-occurrence
SYNE1	*VIP*	<0.001	Co-occurrence
COL16A1	*TFAP2E*	<0.001	Co-occurrence
ATAT1	*NEURL2*	<0.001	Co-occurrence
PAGE1	*IL6R*	<0.001	Co-occurrence
S100A16	*PLA2G4A*	<0.001	Co-occurrence
DMBX1	*ATAT1*	<0.001	Co-occurrence
